# Supplementary material for: Views and experiences of young people on using mHealth platforms for sexual and reproductive health services in rural low-and middle-income countries: A qualitative systematic review
Source: PLOS Digit Health. 2024 Dec 4;3(12):e0000362. doi: 10.1371/journal.pdig.0000362 (PMC11616881; doi:10.1371/journal.pdig.0000362)
Supplement: S1 Table — (DOCX) [file pdig.0000362.s001.docx]

S1 Table. Search strategies tailored to Cochrane Library, Scopus, CINAHL and PsychoINFO

| **Cochrane Library** | |
| --- | --- |
| 1 | Trials matching young adult* OR youth* OR adolescent* OR young people* OR youth population* OR young wom?n* OR young girl* OR young boy* OR young m?n* OR young women* emerging adult* OR adolescent girl* OR adolescent boy* OR adolescen |
| 2 | In ‘Title Abstract Keyword’ AND reproductive health* OR sexual health* OR HIV* OR contraception* OR contraceptives* OR modern contraception* OR contracept service* OR contracept educat* OR contracept counsell |
| 3 | In ‘Title Abstract Keyword’ OR Healthcare providers* OR Healthcare professionals* OR health provider* OR health counsellor* OR health educator |
| 4 | In ‘Title Abstract Keyword’ AND mobile health* OR mHealth* OR mobile phone health technology* OR mobile phone health* OR digital mobile health* OR digital mobile phone health |
| 5 | In ‘Title Abstract Keyword’ AND low-income countries* OR low-and-middle-income nation* OR low to middle income countries* OR middle-income countr* OR low resource countries |
| 6 | In ‘Title Abstract Keyword’ - with Cochrane Library publication date Between Jan 2000 and Nov. 2023, in Trials with 'Public Health' in Cochrane Groups (word variations have been searched) |
| **Scopus** | |
| 1 | (TITLE-ABS-KEY (low-income AND countries* OR low-and-middle income AND nation* OR low AND toAND middle AND income AND countries* OR middle-income AND countr* OR low AND resource AND countries) |
| 2 | TITLE-ABS KEY (mobile AND health* OR mhealth* OR mobile AND phone AND health AND technology* OR mobile AND phone AND health* OR digital AND mobile AND health* OR digital AND mobile AND phone AND health) OR |
| 3 | TITLE-ABS-KEY (healthcare AND providers* OR healthcare AND professionals* OR health AND provider* OR health AND counsellor* OR health AND educator ) OR |
| 4 | TITLE-ABS-KEY (young AND adult* OR youth* OR adolescent* OR young AND people* OR youth AND population* OR young AND wom?n* OR young AND girl* OR young AND boy* OR young AND m?n* OR young AND women* AND emerging AND adult* OR adolescent AND girl* OR adolescent AND boy* OR adolescen) |
| 5 | TITLE-ABS-KEY (reproductive AND health* OR sexual AND health* OR HIV* OR contraception* OR contraceptives* OR modern AND contraception* OR contracept AND service* OR contracept AND educat* OR contracept AND counsell) |
| 6 | AND PUBYEAR > 1999 AND PUBYEAR < 2023. |
| **CINAHL** | |
| S4 | (reproductive health* AND sexual health* AND HIV* AND contraception* AND contraceptives* AND modern contraception* AND contracept service* AND contracept educat* AND contracept counsell* AND young adult* AND youth* AND adolescent* AND young people* AND youth population* AND young wom?n* AND young girl* AND young boy* AND young m?n* AND young women* AND adolescent girl* AND adolescent boy* AND adolescen) OR (healthcare providers* AND healthcare professionals* AND health provider* AND health professional |
| S3 | (reproductive health* OR sexual health* OR HIV* OR contraception* OR contraceptives* OR modern contraception* OR contracept service* OR contracept educat* OR contracept counsel* OR young adult* OR youth* OR adolescent* OR young people* OR youth population* OR young wom?n* OR young girl* OR young boy* OR young m?n* OR young women* emerging adult* OR adolescent girl* OR adolescent boy* OR adolescen |
| S2 | (reproductive health* OR sexual health* OR HIV* OR contraception* OR contraceptives* OR modern contraception* OR contracept service* OR contracept educat* OR contracept counsel* OR young adult* OR youth* OR adolescent* OR young people* OR youth population* OR young wom?n* OR young girl* OR young boy* OR young m?n* OR young women* emerging adult* OR adolescent girl* OR adolescent boy* OR adolescen ) AND ( mobile health* OR mHealth* OR mobile phone health technology* OR mobile phone health |
| S1 | (reproductive health* OR sexual health* OR HIV* OR contraception* OR contraceptives* OR modern contraception* OR contracept service* OR contracept educat* OR contracept counsel* OR young adult* OR youth* OR adolescent* OR young people* OR youth population* OR young wom?n* OR young girl* OR young boy* OR young m?n* OR young women* emerging adult* OR adolescent girl* OR adolescent boy* OR adolescen ) AND ( mobile health* OR mHealth* OR mobile phone health technology* OR mobile phone health* |
|  | limiters - full text; references available; published date: 20000101-20231231; peer reviewed; clinical queries: qualitative - best balance; human; journal subset: public health; geographic subset: Africa; language: English; special interest: men's health, public health, women's health |
| **PsychoINFO** | |
| 1 | (young adult* or youth* or adolescent* or young people* or youth population* or young wom?n* or young girl* or young boy* or young m?n* or young women* emerging adult* or adolescent girl* or adolescent boy* or adolescen).mp. [mp=title, abstract, heading word, table of contents, key concepts, original title, tests & measures, mesh] |
| 2 | (reproductive health* or sexual health* or HIV* or contraception* or contraceptives* or modern contraception* or contracept service* or contracept educat* or contracept counsell).mp. [mp=title, abstract, heading word, table o’f contents, key concepts, original title, tests & measures, mesh] |
| 3 | (healthcare providers* or healthcare professionals* or health provider* or health counsellor* or health educator).mp. [mp=title, abstract, heading word, table of contents, key concepts, original title, tests & measures, mesh] |
| 4 | (mobile health* or mHealth* or mobile phone health technology* or mobile phone health* or digital mobile health* or digital mobile phone health).mp. [mp=title, abstract, heading word, table of contents, key concepts, original title, tests & measures, mesh] |
| 5 | (low-income countries* or low-and-middle-income nation* or low to middle income countries* or middle-income countr* or low resource countries).mp. [mp=title, abstract, heading word, table of contents, key concepts, original title, tests & measures, mesh] |
| 6 | limit to (full text and human and english language and yr="2000 -2023") |
