# Supplementary material for: Views and experiences of young people on using mHealth platforms for sexual and reproductive health services in rural low-and middle-income countries: A qualitative systematic review
Source: PLOS Digit Health. 2024 Dec 4;3(12):e0000362. doi: 10.1371/journal.pdig.0000362 (PMC11616881; doi:10.1371/journal.pdig.0000362)
Supplement: S2 Table — (DOCX) [file pdig.0000362.s002.docx]

S2 Table. Overall summary of assessment of quality and CERQual explanation of findings

| **Components** | **Studies contributing to the assessment findings and ratings** | **Assessment quality ratings** | **Overall CERQual**  **assessment quality ratings of evidence** | **Explanation of overall CERQual assessment of quality findings** |
| --- | --- | --- | --- | --- |
| **Methodological limitations** | All 26 studies (29-54) included in this review were rated as no or very minor concerns overall, with 7 having minor issues as detailed below:   1. Unclear of ethical procedures, 3 studies (32, 37, 46). 2. Unclear reflexivity issues, 3 studies (32, 40, 49). 3. Unclear data analysis procedure, 1 study (46). | Minor concerns | High Quality | Overall, the finding was graded as high confidence as it is highly likely that the finding is a reasonable representation of young people’s experiences after using mobile phone-based platform interventions regarding SRH information and services.    All 26 studies (29-54) included in this review were rated as no or very minor concerns overall with 7 having minor issues as detailed below:  For methodological limitations:   1. Unclear of ethical procedures, 3 studies (32, 37, 46). 2. Unclear reflexivity issues, 3 studies (32, 40, 49). 3. Unclear data analysis procedure, 1 study (46) 1 study.   For data adequacy, 3 studies (31, 38, 52) had minor data issues.  For coherence and relevance, both were rated no or very minor concerns. |
| **Coherence** | All 26 studies (29-54) included in this review were rated as no or very minor concerns overall. | Minor concerns |  |  |
| **Adequacy** | All 26 studies (29-54) included in this review were rated as no or very minor concerns overall with 3 studies (31, 38, 52) having minor data issues. | Minor-moderate concerns |  |  |
| **Relevance** | All 26 studies (29-54) included in this review were rated as no or very minor concerns overall. | Minor concerns |  |  |
