# Supplementary material for: Views and experiences of young people on using mHealth platforms for sexual and reproductive health services in rural low-and middle-income countries: A qualitative systematic review
Source: PLOS Digit Health. 2024 Dec 4;3(12):e0000362. doi: 10.1371/journal.pdig.0000362 (PMC11616881; doi:10.1371/journal.pdig.0000362)
Supplement: S3 Table — (DOCX) [file pdig.0000362.s003.docx]

S3 Table. Assessment of studies under each CERQual component

| **Studies** | **Assessment of methodological limitations** | **Assessment of relevance** | **Assessment of data adequacy** | **Assessment of coherence** | **CERQual Assessment of quality** |
| --- | --- | --- | --- | --- | --- |
| **Evelia et al 2015** | Minor concerns | No or very minor concerns | No or very minor concerns | No or very minor concerns | High |
| *Notes* | *Reflexivity not clear from reading paper.*  *Ethical issues not clearly described.* |  |  |  |  |
| **Smith et al 2017** | No or very minor concerns | No or very minor concerns | No or very minor concerns | No or very minor concerns | High |
| **Dev et al 2019** | Minor concerns | No or very minor concerns | No or very minor concerns | No or very minor concerns | High |
| *Notes* | *Reflexivity not clear from reading paper.* |  |  |  |  |
| **Blanc et al 2016** | Minor concerns | No or very minor concerns | No or very minor concerns | No or very minor concerns | High |
| *Notes* | *Reflexivity not clear from reading paper.*  *Ethical issues not clearly described.* |  |  |  |  |
| **Jamison et al 2013** | Minor concerns | No or very minor concerns | No or very minor concerns | No or very minor concerns | High |
| *Notes* | *Ethical issues not clearly described.* |  |  |  |  |
| **Parajuli & Doneys 2017** | No or very minor concerns | No or very minor concerns | No or very minor concerns | No or very minor concerns | High |
| **Ybarra et al 2020** | No or very minor concerns | No or very minor concerns | No or very minor concerns | No or very minor concerns | High |
| **Winskell et al 2018** | No or very minor concerns | Minor concerns | No or very minor concerns | No or very minor concerns | High |
| **Visser et al 2020** | No or very minor concerns | Moderately rich data | No or very minor concerns | No or very minor concerns | High |
| **Sabben et al 2019** | Minor concerns | No or very minor concerns | No or very minor concerns | No or very minor concerns | High |
| *Notes* | *Reflexivity not clear from reading paper.* |  |  |  |  |
| **McCharty et al 2018** | No or very minor concerns | No or very minor concerns | No or very minor concerns | No or very minor concerns | High |
| **Adeagbo et al 2019** | No or very minor concerns | No or very minor concerns | No or very minor concerns | No or very minor concerns | High |
| **Akinfaderin-Agarau et al 2012** | No or very minor concerns | No or very minor concerns | No or very minor concerns | No or very minor concerns | High |
| **Merrill et al 2018** | No or very minor concerns | No or very minor concerns | No or very minor concerns | No or very minor concerns | High |
| **Nigatu et al 2017** | Minor concerns | No or very minor concerns | No or very minor concerns | No or very minor concerns | Moderate |
| *Notes* | *Data analysis procedure not clear from reading the paper.*  *Ethical issues not clearly described in detail.* |  |  |  |  |
| **FHI 360 et al 2013** | Minor concerns | Minor concerns | No or very minor concerns | No or very minor concerns | Moderate |
| *Notes* | *Reflexivity does not clear from reading paper.*  *Ethical issues not clearly described.* | *Moderately rich data* |  |  |  |
| **Ong et al 2020** | No or very minor concerns | No or very minor concerns | No or very minor concerns | No or very minor concerns | High |
| **Guerrero et al 2020** | No or very minor concerns | No or very minor concerns | No or very minor concerns | No or very minor concerns | High |
| **Girl Effect and Women Deliver 2020** | No or very minor concerns | No or very minor concerns | No or very minor concerns | No or very minor concerns | High |
| **Memiah et al 2014** | No or very minor concerns | No or very minor concerns | No or very minor concerns | No or very minor concerns | High |
| **Laidlaw et al 2017** | No or very minor concerns | No or very minor concerns | No or very minor concerns | No or very minor concerns | High |
| **L’Engle et al 2013** | No or very minor concerns | Minor concerns | No or very minor concerns | No or very minor concerns | High |
| *Notes* |  | *Moderately rich data* |  |  |  |
| **Ampt et al 2020** | No or very minor concerns | Minor concerns | No or very minor concerns | No or very minor concerns | High |
| *Notes* |  | *Moderately rich data* |  |  |  |
| **Duclos et al 2017** | No or very minor concerns | No or very minor concerns | No or very minor concerns | No or very minor concerns | High |
| **Eckersberger et al 2017** | No or very minor concerns | No or very minor concerns | No or very minor concerns | No or very minor concerns | High |
| **Vahdat et al 2013** | No or very minor concerns | No or very minor concerns | No or very minor concerns | No or very minor concerns | High |
