# Supplementary material for: Views and experiences of young people on using mHealth platforms for sexual and reproductive health services in rural low-and middle-income countries: A qualitative systematic review
Source: PLOS Digit Health. 2024 Dec 4;3(12):e0000362. doi: 10.1371/journal.pdig.0000362 (PMC11616881; doi:10.1371/journal.pdig.0000362)
Supplement: Attachment — Submitted filename: 2023 .docx [file pdig.0000362.s004.docx]

**PLOS Digital Health Journal**

**Reviewer comments on:** Views and experiences of young people on using mHealth platforms for sexual and reproductive health services in rural low- and middle-income countries: a qualitative systematic review

**Response to reviewer comments**

| **Reviewers Comments** | **Response to reviewers’ comments** | **Changes made** | **Location of changes in the manuscript text** | **Changes made pages in the manuscript text** |
| --- | --- | --- | --- | --- |
| **Reviewer 1** |  |  |  |  |
| In the PROSPERO protocol, the research question is “How effective are mHealth communication platforms/channels for accessing sexual and reproductive health information and services by young people in rural settings in LMICs?” (CRD42020199221) whereas in the manuscript the authors “synthesized the current evidence to provide a comprehensive understanding of the views and experiences of young people in rural areas using mobile platforms for SRH information and services in rural and remote areas in LMICs.” The authors should update the protocol and provide a rationale for the change of focus from effectiveness to experiences in the manuscript. They should also state the current research question more clearly at the end of the introduction. | Thank you for drawing our attention to the difference between the research question of the protocol and the manuscript.  The rational for the change of the research question was altered to agree with the qualitative nature of the study. This occurred because during the review process it became apparent that there were insufficient studies that could be included to evaluate efficacy. | The difference between of the research question between the PROSPERO protocol, and manuscript is updated in the protocol.  The current research question is now clearly stated at the introductory section of the manuscript | No changes made.  The change is highlighted in green. | Page 4 |
| The PROSPERO protocol lists the following databases for the searches: MEDLINE, Embase, PsycINFO and Scopus. In the manuscript an additional database is listed (CINAHL). The authors should revise the protocol and explain all deviations from the original plan in the manuscript. | Thank you for drawing our attention to the differences between the databases of the protocol and manuscript. | We will revise the protocol to include the additional database (CINAHL). | The amendment will be effected in the protocol. | N/A |
| Regarding population of interest, the authors only mention boys/men and girls/women (page 9). A rationale for not including more genders, such as transgender and non-binary, should be provided – and discussed as a limitation to the study in the discussion.  The logic behind only specifying “Africa” and “sub-Saharan Africa” in the search terms should also be given.  Thirdly, the target age range was 10-24 (page 9), but it is clear from Table 5 that the authors have included studies with older participants: - Vahdat: 19-29 years - Ducleos: age range? - Smith: 22 years & above - Jamison: 18-35 - Parajuli & Doneys: 16-36 - McCharty: 15-30 - Adeagbo: 18-34 - Akinfaderin-Agarau: 12-30 - Ampt: 16 years or older - FHI 360 USAID (?): 29 years or younger - Memiah: 14-29 years - Eckersberger: 18 & above - L’engle: 19-29 years | Thank you for drawing our attention to these important issues on gender, study settings, and age range of participants.  Thank you for your useful comment on the logic behind only specifying “Africa” and “sub-Saharan Africa” in the search terms should also be given.  Please, Africa and sub-Saharan Africa are part of LMICs. They were used as search terms with “low-income countries” “low- and-middle-income nation” “low to middle income countries” “middle-income countries” “developing countries” “low resource countries” | Not including more genders, such as transgender and non-binary is indicated as a limitation of the study.  The reason for specifying “Africa” and “sub-Saharan Africa as a geographical setting was to make the search comprehensive and to also capture more studies in LMICs especially studies which have used sub-Saharan Africa and Africa in their title headings.  The samples including older participants above or outside the age range of interest (10-24) were studies where the data could be disaggregated. An explanation is indicated in the manuscript. | The changes are highlighted green in the manuscript text.  No changes made.  The further  explanation is highlighted in green in the text.  A footnote explanation is provided at table 5 relating to the specific age groups of 10-24 years. | Page 31.  Page 7  Page 18 |
| I also suggest that the authors sort the included articles in Table 5 in alphabetical order and provide year of publication after respective authors' names.  Furthermore, the results column is framed as “Key findings on barriers for young people in accessing SRH health”, which only partly reflects the research question (views and experiences) | Thank you for your suggestion on sorting respective authors in alphabetical order with publication year.  Thank you for drawing our attention to the key findings partly reflecting the research question. | The authors of the articles in Table 5 are now sort in alphabetical order with the year of publication after respective authors' names.  The key findings title is revised in table 5 to reflect the views and experiences of young people. | The amendment is highlighted in green in the title.  The amendment is highlighted in green in the title. | Pages 11 to 18.  Pages 11 to 18. |
| Last but not least, the search was restricted to the years 2000-2020. It is of utmost importance that the authors include potential studies published between 2020 up until today, especially considering the rapid development of mHealth interventions, why the authors must update the search. | Thank you for your useful comment and suggestion. | Please, we have re-run the databases from 2020 to 2023 current. However, no new papers were found. | The information is updated throughout the manuscript text highlighted in green. | Pages1 to 46. |
| **Reviewer 2** |  |  |  |  |
| A rigorous, and well written paper was submitted to the journal. The authors abided to the requirements of a qualitative systematic review. I cannot fault them for this paper in terms of method or writing.  **Reviewer 3** | Thank you for your observation. |  |  |  |
| The authors have successfully examined several important factors impacting access to SRH services among young individuals in LMICs, including cultural context, financial barriers, and privacy and confidentiality concerns by synthesizing available evidence. If possible, another aspect that might be worth discussing is the role of parental or major financial supporters on young people's access to SRH services, given their potential impact in either facilitating or hindering access. These individuals often play an important role either by providing the necessary financial resources for obtaining a phone and services or by imposing constraints, such as sharing a phone among family members, which could potentially compromise confidentiality, as pointed out in the review. | Thank you for your suggestion for including parental roles as major financial supporters either facilitating or hindering young people access to SRH services. | This suggestion is captured under general barriers for use of mobile platforms SRH information and services by participants in Table 6.  The result is also updated under barriers to using mHealth platforms SRH services in text. | The additional information is highlighted in green in table 6.  The additional information is highlighted in green. | Pages 22 and 23.  Pages 25 |
| Moreover, the cultural context plays an essential role, as parents may hold reservations about sexual contact among this population due to religious or cultural reasons, potentially leading to concerns that if parents were aware of such activities, they might resort to actions that can influence young people's decision-making regarding SRH services. | Thank you for drawing our attention to this important information. | This suggestion is captured under barriers under social norms and beliefs as additional information in Table 6.  The discussion section is also updated. | The additional information is highlighted in green.  The additional information is highlighted in green. | Page 23.  Page 27. |
