# Supplementary material for: Views and experiences of young people on using mHealth platforms for sexual and reproductive health services in rural low-and middle-income countries: A qualitative systematic review
Source: PLOS Digit Health. 2024 Dec 4;3(12):e0000362. doi: 10.1371/journal.pdig.0000362 (PMC11616881; doi:10.1371/journal.pdig.0000362)
Supplement: Attachment — Submitted filename: Resonse to Reviewers comments -24-08-24.docx [file pdig.0000362.s005.docx]

# Journal: PLOS Digital Health.

Title of Manuscript: Views and experiences of young people on using mHealth platforms for sexual and reproductive health services in rural low- and middle-income countries: a qualitative systematic review (PDIG-D-23-00332R1)

**Response to reviewers’ comments**

| **Reviewers Comments** | **Response to reviewers’ comments** | **Changes made** | **Location of changes/additions in track changes highlighted red in the manuscript text** | **Changes/additional made pages/line numbers in the manuscript text** |
| --- | --- | --- | --- | --- |
| **Reviewer 4** |  |  |  |  |
| **General comments** |  |  |  |  |
| The English grammar in the methods and discussion sections of the paper has been revised | Thank you for this important observation and suggestion. | The corrections of grammar issues. | The changes made are in the track changes. |  |
| Methods line 100: The added sentence in green here is completely unnecessary and should be removed. Stating that "Studies were limited to those published in English from 2000-2023 given..." is perfectly sufficient. | Thank you for this important suggestion. | The unnecessary additional sentence has been deleted. | The changes made are in the track changes. | Line 110-page 5 |
| Methods line 122 : The sentence that starts with "Also, in studies where" should be "Studies in which the ages of the participants were outside this range but could be segregated were included." | Thank you for this important suggestion. | The sentence has been revised as suggested. | The changes made are in the track changes. | Line 127-page 6 |
| Table 5: "et al" should be "et al." That is et al. always ends with a period. | Thank you for this important suggestion. | Periods have been introduced to et al as suggested. | The changes made are in the track changes. | Page 10-16. |
| Results line 29: "partners for young women" should be "partners of young women" | Thank you for this important suggestion. | The word for has been replaced by of. | The changes made are in the track changes | Line 31 -page 25 |
| Results line 17: This sentence is very awkwardly phrased and took me several rereads to understand what was being said. | Please, thank you for drawing our attention. | The sentence has been revised for understanding. | The changes made are in the track changes | Lines 17-21-page 24 |
| Discussion line 62 : missing commas, should be "electricity, connectivity, and coverage" | Thank you for drawing our attention to this. | The missing commas have been inserted. | In track changes**.** | Line 65-page 26. |
| Discussion line 93: missing comma between Bolivia and Palestine | Thank you for drawing our attention to this. | The comma has been inserted. | In track changes. | Line 97-page 27. |
| Discussion line 95: "religion" should be "religious" | Thank you for this important suggestion. | The word "religion" has been changed to “religious" | In track changes. | Line 99-page 27. |
| Discussion line 116 : "non android" should be "non-Android" | Thank you for this important suggestion. | The word “non android" has been changed to “non-Android". | In track changes. | Line 122-page 28. |
| Discussion line 126 rather 140: sentence starting with "Preference for voice messaging..." has several grammatical mistakes. "to use" (line 127) doesn't seem grammatically correct, and "the to the" (line 128) seems like a typo/a word is missing. | Thank you for this important observation. | The sentence is edited to strengthen the grammar. | In track changes | Line 139, page 29 |
| Discussion line 138: "Whiles" should be "While" | Thank you for this important observation. | The word “Whiles" has been replaced with “While" | In track changes**.** | Lines 147&149, page 29 |
| Discussion line 130 instead of 144: The sentence that begins with "The findings indicated that" seems to be redundant with the entire next paragraph (lines 129-141), and I'm not sure why this redundancy is needed. This sentence is also a bit difficult to parse since there's so much information contained in it, personally I'd recommend getting rid of this sentence since as far as I can tell all its information is contained in the following paragraph. If the authors want to keep the sentence, I'd make it clearer why the redundancy with the next paragraph is needed or rewrite the sentence to reduce redundancy. | Thank you for this important observation. | The entire redundant sentence has been deleted. | N/A | Page 29. |
| Discussion line 143: what does "familiar among them in their settings" mean? This should be rephrased for clarity | Thank you for this important suggestion | The sentence has been rephrased. | In track changes**.** | Line 154, page 29. |
| Discussion line 153: The green line here is just not grammatically correct. Also, it is a limitation, the sentence shouldn't say it "could be" a limitation. | Thank you for this important suggestion | Please, the grammar has been corrected. | In track changes. | Line 232, page 30. |
| Discussion line 151: Shouldn't this be 2000-2023? | Thank you for this important suggestion | We have edited to be 2000-2023. | In track changes. | Line 162, page 29. |
| Discussion line 156: there is no need to mention the strength of the study | Thank you for this important suggestion | The phrase, the strengths of the study has been deleted. | N/A | Line 219, page 30. |
| Acknowledgments line 182: Shouldn't use "my" in a research paper | Thank you for this important suggestion | The word “my” has been deleted. | N/A | Page 31. |
|  |  |  |  |  |
| Reviewer 5 |  |  |  |  |
| While the introduction does a good job of setting the context, it could benefit from a brief mention of any significant advancements or changes in mHealth interventions post-2020, given the rapid evolution of technology | Thank you for this important suggestion | The suggestion has been addressed in the introduction. | In track changes**.** | Page 4. |
| It would be beneficial to elaborate on the role of socio-cultural norms and parental influence, as these are significant barriers. Discussing the potential impact of these factors on the uptake of mHealth services can provide deeper insights. | Thank you for this important suggestion | The suggestion has been addressed in the introduction. | In track changes. | Page 3. |
