# Supplementary material for: Views and experiences of young people on using mHealth platforms for sexual and reproductive health services in rural low-and middle-income countries: A qualitative systematic review
Source: PLOS Digit Health. 2024 Dec 4;3(12):e0000362. doi: 10.1371/journal.pdig.0000362 (PMC11616881; doi:10.1371/journal.pdig.0000362)
Supplement: Attachment — Submitted filename: A Rebuttal letter-PLOS DIGITAL HEALTH -Journal-01-10-24.docx [file pdig.0000362.s006.docx]

# Rebuttal Letter.

# Journal: PLOS Digital Health.

Title of Manuscript: Views and experiences of young people on using mHealth platforms for sexual and reproductive health services in rural low- and middle-income countries: a qualitative systematic review (PDIG-D-23-00332R2)

**Response to reviewers’ comments**

| **Reviewers Comments** | **Response to reviewers’ comments** | **Changes made** | **Location of changes** | **Changes/additional made pages/line numbers in the manuscript text** |
| --- | --- | --- | --- | --- |
| **Discussion** |  |  |  |  |
| Discussion Line 63: "The review finding reported..." should be "The review findings reported" | Thank you very much for the grammatical review and suggestion. | The suggested change has been made. | The addition made is in track changes highlighted red in the manuscript text. | Line 63-page 26 |
| Discussion Line 64: "...as a barriers to use of..." should be "...as barriers to the use of..." | Thank you very much for the grammatical review and suggestion. | The suggested grammatical change has been made. | The deletion is made in the manuscript text. | Line 64-page 26 |
| Discussion Line 113: "The ability to engage young people with wide range..." should needs an "a" before "wide range" | Thank you very much for the grammatical review and suggestion. | “a” has been added before “wide range”. | The addition is in track changes highlighted red in the manuscript text. | Line 113-page 28 |
| Discussion Line 126-127: peoples should be possessive, so this should be "...rural young peoples' preference..." | Thank you very much for the grammatical review and suggestion. | Rural young peoples' preference is now made possessive. | The addition is in track changes highlighted red in the manuscript text. | Line 126-page 29 |
